# Aqueous cytokine and chemokine analysis in uveitis associated with tuberculosis

**Published:** 2012-03-02

**Authors:** Marcus Ang, Gemmy Cheung, Maya Vania, Jinmiao Chen, Henry Yang, Jing Li, Soon-Phaik Chee

**Affiliations:** 1 Singapore National Eye Centre, Singapore; 2 Singapore Eye Research Institute, Singapore; 3 Singapore Immunology Network, Singapore; 4 Department of Ophthalmology, National University Health Systems, Singapore; 5 Department of Ophthalmology, Xin Hua Hospital, Shanghai Jiao Tong University School of Medicine, Shanghai, China

## Abstract

**Purpose:**

The aim of this study was to study the aqueous cytokine and chemokine composition in patients with uveitis associated with tuberculosis (TAU).

**Methods:**

We present a prospective case series of consecutive new patients with active uveitis presenting at a single tertiary center (January 1, 2008-January 1, 2010). Patients with no ocular pathology other than cataracts were enrolled as non-inflammatory controls. Aqueous samples were taken from all study subjects and analyzed using a magnetic color-bead-based multiplex assay for cytokine and chemokine concentrations.

**Results:**

Twenty-five eyes of 25 patients with active uveitis with suspected tuberculosis (TB) and 23 non-inflammatory controls were enrolled. Ten patients tested positive on a tuberculin skin test and interferon-gamma release assay; all ten patients responded to anti-TB treatment with no recurrences (TAU). The remaining 15 eyes were negative for the above tests and had no other underlying causes for uveitis found on clinical evaluation and investigations; therefore, they were classified as “idiopathic uveitis” (IU). The TAU group showed significantly higher levels of interleukin-6 (IL-6; p=0.047), interleukin-8 (CXCL8; p=0.001), monokine induced by interferon-gamma (CXCL9; p=0.001), and interferon-gamma-induced protein 10 (IP-10 or CXCL10; p=0.002), compared to the controls. The IU group showed significantly higher levels of IL-6 (p=0.008), monocyte chemotactic protein-1 (CCL2; p=0.036), CXCL8 (p=0.001), and IL-9 (p=0.045), and significantly lower levels of IL-2 (p=0.011), IL-12 (p=0.001), and tumor necrosis factor (TNF)-α (p=0.001), compared to the controls. Heat map analysis revealed significant differences in aqueous cytokine and chemokine concentrations among the TAU patients, the IU patients, and the controls.

**Conclusions:**

In our study population, aqueous cytokine and chemokine analyses suggest that subjects with uveitis associated with TB who respond to anti-TB therapy do not have an active ocular tuberculous infection, but rather an autoimmune-related ocular inflammation that may be triggered by TB.

## Introduction

Uveitis is a sight-threatening disease that accounts for more than 10% of blindness worldwide [1]. While uveitis may be associated with infections or autoimmune or systemic diseases, up to 60% of all patients have no underlying etiology detected, and the uveitis is thus labeled “idiopathic” [1-3]. In Singapore, a developed country with an intermediate burden of tuberculosis (TB), the association of TB with idiopathic uveitis is controversial [4,5]. Although no disease other than manifest or latent TB is found, the diagnosis of ocular TB cannot be confirmed, as there is often no evidence of *Mycobacterium tuberculosis* from their ocular samples [6]. The detection of *Mycobacterium tuberculosis* is rare [7], as methods such as culture on Lowenstein Jenson agar, acid-fast bacilli (AFB) smear, and demonstration of *Mycobacterium tuberculosis* nucleic acid using polymerase chain reaction (PCR) have low sensitivities [8]. Thus, diagnoses of uveitis associated with TB (TAU) are mostly presumptive, i.e., made with a positive tuberculin skin test (TST), a positive interferon-gamma release assay (IGRA) test, findings suggestive of previous pulmonary TB on chest X-ray (CXR), and/or concomitant active extraocular TB infection [4,6,9,10].

The aqueous humor is important for the maintenance of both physiologic function and metabolic homeostasis within the anterior chamber [11]. Components of the aqueous humor are a reflection of the state of intraocular tissues, and they change rapidly with the development or progression of disease [12]. Abnormal concentrations of various cytokines have been reported in the aqueous associated with different forms of uveitis [13-20], and distinct cytokine profiles may be used to identify specific diseases [15].

In this pilot study, we compared the aqueous cytokine and chemokine compositions in patients with TAU and no other causes of inflammation to the aqueous from patients with no ocular inflammation, as well as aqueous from patients with idiopathic uveitis with no evidence of TB or other diseases.

## Methods

We conducted a prospective, comparative case series of consecutive patients with active uveitis presenting at the Singapore National Eye Centre Ocular Inflammation and Immunology Service, from January 1, 2008, to January 1, 2010. The study was approved by the local Institutional Review Board and performed in accordance with the tenets of the Declaration of Helsinki. Informed consent was obtained from all subjects. All new patients who present to our facility with uveitis undergo a systemic and clinical examination, with investigations as previously described [4]. In this study, we noted patient demographics, clinical history, and features of uveitis, using the criteria set by the Standardization of Uveitis Nomenclature (SUN) working group for scoring the anatomic location, onset, duration, course, and activity [21]. Patients with no ocular pathology other than cataract were enrolled as non-inflammatory controls.

All subjects underwent investigations, including complete blood count, erythrocyte sedimentation rate, liver enzyme panel, infectious disease screen (which included Venereal Disease Research Laboratory and enzyme immunoassay test for syphilis, TST, urine microscopy), and chest X-ray. Other tests were performed as well, such as human leukocyte antigen (HLA) B27 screen, IGRA tests, AFB smear from throat swabs, and PCR assays for TB DNA from aqueous humor. As part of our routine investigations, TST was performed using intradermal injection of 0.1 ml (2 tuberculin units) purified protein derivative (RT23 SSI–2T.U. /0.1 ml Statens Serum Institut, Copenhagen, Denmark) [22]. The resultant induration was measured at 72 h with a ruler by an independent observer, and it was considered positive if ≥15 mm (as validated in our population) [23]. IGRA tests such as QuantiFERON-TB Gold In-Tube (QFT; Cellestis Inc., Carnegie, Australia) and T-SPOT.TB (Oxford Immunotec, Abingdon, UK) were performed according to guidelines, and blood samples taken before each TST was performed [24,25]. Uveitis associated with TB (TAU) was defined as a positive TST with a positive IGRA and no other cause found to contribute to the ocular inflammation, as well as response to anti-TB therapy [26,27].

### Aqueous humor sampling

Before any treatment was started, aqueous humor was sampled from the eye with more severe active inflammation, using a described technique [28]. Briefly, we performed the procedure under topical anesthesia, using an aseptic technique: a 27-gauge needle attached to an insulin syringe was inserted at the peripheral clear cornea in a plane above and parallel to the iris. Under direct vision aided by a slit-lamp, approximately 100 µl of aqueous fluid were withdrawn. All patients were re-examined at 30 min and one week after the procedure, with topical antibiotics prescribed for three days. Aqueous humor was also sampled from the non-inflammatory controls under an operating microscope, using the technique described, just before commencing cataract surgery. Aqueous samples were immediately spun at 300× g for five min at 4 °C, using a tabletop microcentrifuge, and stored at –80 °C until analysis.

### Cytokine measurement

Bio-Plex Pro™ magnetic color-bead-based multiplex assay (Bio-Rad Laboratories, Inc., Hercules, CA) was used to measure aqueous cytokine concentrations. Thirty-five microliters (35 µl) of each aqueous humor sample were used. Fluorescence intensity (FI) from the immunoassay was acquired and analyzed using the Bio-Plex™ 200 System (software version 6.0; Bio-Rad Laboratories, Inc.).

### Statistical analysis

Statistical analysis was performed using Statistica Version 6.0 (Statsoft, Tulsa, OK). Statistical significance was set at p<0.05. For categorical variables, frequency distribution and percentages were calculated. Comparisons of baseline characteristics were conducted by Fisher’s exact tests. To compare the differences in cytokine concentrations, a Mann–Whitney U test with Bonferroni correction was performed. In addition, multivariate binary logistic regression analysis was performed to adjust for the differences in age, gender, and ethnic group. The two-tailed, nonparametric Spearman method was used to assess for correlations between variables.

### Classification analysis

Step-wise analysis was performed to generate cytokine profiles to best separate the different disease groups. For each cytokine, the measured concentration was logarithmically transformed. An initial analysis was used on all available cytokines and chemokines, and then it was refined to include those that showed significant differences on two-sample Student’s *t*-tests. The panel of differentially expressed cytokines was then further reduced by the removal of highly correlated cytokines. A separate decision tree analysis was performed to step-wise select marker cytokines, using open-source data mining software WEKA. Fivefold cross validation was conducted to examine the error rate of this method.

## Results

### Patient demographics and clinical findings

We enrolled 25 new patients who presented with acute, active uveitis, and who were suspected of having TB upon initial clinical examination, with clinical signs consistent with TAU, such as granulomatous inflammation, broad-based posterior synechiae, or retinal vasculitis, with or without choroiditis [29,30] Based on the diagnostic criteria above, ten patients had TAU, all of whom responded to anti-TB therapy. These patients had a positive QFT (n=9), a positive T-SPOT.TB (n=5), or both positive QFT and T-SPOT.TB (n=4); all had TST indurations ≥15 mm (range 15–35 mm). After complete clinical evaluations and investigations, it was determined that the other 15 subjects with uveitis had no underlying cause or disease, including negative TST (TST<10 mm) and negative IGRA, with no other etiology found from the investigations described above; as such, they were defined as having idiopathic uveitis (IU). Patient demographic data are presented in Table 1.

**Table 1:** Demographic features of patients.

		**Groups**			
**Characteristics**	**All (n=48)**	**TAU (n=10)**	**IU (n=15)**	**Controls (n=23)**	p-value*
**Age (mean±SD)**	**57.8±18.4**	**41.1±20.9**	**55.8±13.7**	**66.3±15.0**	**0.001**
**Gender (%)**					
Male	21 (43.8)	4 (40.0)	6 (40.0)	11 (48.0)	0.678
Female	27 (56.3)	6 (60.0)	9 (60.0)	12 (52.0)	
**Race (%)**					
Chinese	32 (66.7)	2 (20.0)	11 (73.3)	19 (82.6)	0.015
Malay	8 (16.7)	3 (30.0)	3 (20.0)	2 (8.6)	
Indian	4 (8.3)	2 (20.0)	1 (6.7)	1 (4.4)	
Others	4 (8.3)	3 (30.0)	0 (0.0)	1 (4.4)	

TAU: Uveitis associated with tuberculosis; IU=Idiopathic uveitis; SD=Standard Deviation. *p-value was obtained from one-way ANOVA test or Pearson χ^2^ comparing TAU, IU and non-inflammatory controls.

All uveitis patients had active inflammation with SUN grade 1+ (n=10) or 2+ (n=16) anterior chamber cells at the time of aqueous sampling. The patients had predominantly anterior (n=17), intermediate (n=2), posterior (n=3), or panuveitis (n=4). There were no cases of choroiditis. Only four patients had bilateral disease; three of those had panuveitis and one had anterior uveitis. Aqueous PCR analysis was performed for *Mycobacterium tuberculosis*, cytomegalovirus, herpes simplex virus, varicella-zoster virus, rubella virus, and toxoplasmosis genomic DNA; the results were negative for all samples.

Twenty-three patients who consulted for cataract surgery were enrolled in the study as non-inflammatory controls. The controls had no known systemic inflammatory, autoimmune, or immunosuppressive disease, as well as no pre-existing ocular disease or previous ocular surgery.

### Aqueous humor cytokine concentration

Table 2 describes the concentrations of interleukin (IL)-2, interferon-gamma (IFN-γ), IL-6, IL-10, IL-12, tumor necrosis factor alpha (TNF-α), monocyte chemotactic protein-1 (CCL2), IL-8 (also known as CXCL8), monocyte induced by interferon-gamma (also known as CXCL9 or IL-9), interferon-gamma induced protein 10 (IP-10, CXCL-10), and IL-9 in aqueous samples from the TAU, IU, and control groups. Calculated concentrations equal to or lower than the low detection limit of the analysis for individual cytokine were taken as non-measurable. By univariate analysis, the TAU patients showed significantly higher concentrations of IL-6 (p=0.047), CXCL8 (p=0.001), CXCL9 (p=0.001), and CXCL10 (p=0.002), compared to the non-inflammatory controls. However, when they were adjusted for age and gender differences, only CXCL8 (p=0.043) showed significant differences between the TAU patients and the controls (p=0.071 for IL-6, p=0.175 for CXCL9, p=0.107 for CXCL10).

**Table 2:** Aqueous cytokine concentrations comparing TAU, IU, and Controls.

** **	**TAU n=10**			**IU n=15**			**Controls n=23**			** **
**Cytokines**	**Median**	**Range (%)**	**Mean±Std error**	**Median**	**Range (%)**	**Mean±Std error**	**Median**	**Range (%)**	**Mean±Std error**	**Sensitivity**
IL-2	3.4*	0–5 (9/10)	3.0±0.4	0.0‡	0–9 (2/15)	0.9±0.6	2.8	0–17 (17/23)	3.4±0.8	1.6
IFN-γ	26.3	0–219 (8/10)	66.9±24.5	10.9	0–71 (8/15)	17.3±5.9	12.7	0–91 (12/21)	15.6±4.5	6.4
IL-6	80.7†	2–2018 (7/9)	470.4±276.8	7.0‡	1–68 (11/13)	21.8±6.1	2.2	0–14 (8/22)	3.4±0.6	2.6
IL-10	1.9	0–4 (9/10)	2.0±0.4	0.3	0–8 (7/15)	1.3±0.6	1.5	0–5 (20/23)	1.7±0.2	0.3
IL-12	7.3*	2–15 (7/10)	7.1±1.4	0.0‡	0–2 (0/15)	0.5±0.2	8.7	0–27 (19/23)	8.7±1.3	3.5
TNF-α	12.1*	1–23 (8/10)	12.0±2.6	0.0‡	0–4 (0/14)	0.5±0.3	9.6	0–34 (17/23)	9.8±1.7	6.0
CCL2	380.8	158–1124 (10/10)	485.3±98.8	531.3‡	47–1354 (15/15)	604.7±113.9	180.9	32–962 (23/23)	252.5±50.5	1.1
CXCL8	61.7†	4–538 (10/10)	104.9±50.3	17.6‡	4–135 (15/15)	39.6±12.1	3.0	1–20 (21/22)	4.8±1.0	1.0
CXCL9	4790.6*†	81–51068 (9/9)	17115.9±6393.3	0.0	0–401 (5/11)	102.7±42.6	63.0	0–834 (19/21)	153.3±44.8	1.2
CXCL10	15621.1†	41–114173 (10/10)	49766.9±17640.0	631.6	0–107547 (11/15)	18632.6±9548.9	56.3	0–766 (20/22)	147.9±45.7	6.1
IL-9	8.26†	4–18 (9/9)	8.8±1.4	15.6‡	0–45 (9/10)	16.5±4.0	5.5	0–11 (17/19)	5.5±0.6	2.5

TAU=uveitis associated with tuberculosis, IU: idiopathic uveitis; IFN-γ: interferon-gamma; TNF-α: tumor necrosis factor-alpha, CCL2: MCP-1, monocyte chemotactic protein-1; CXCL8 (IL-8, interleukin-8); CXCL9 (MIG, monokine induced by interferon-gamma); CXCL10 (IP-10, interferon-gamma-induced protein 10). Cytokines level at pg/ml. * p<0.05, for TAU versus IU; †p<0.05, for TAU versus controls; ‡ p<0.05, for IU versus controls by Mann-Whitney U test with Bonferroni correction.

**Figure 1:**
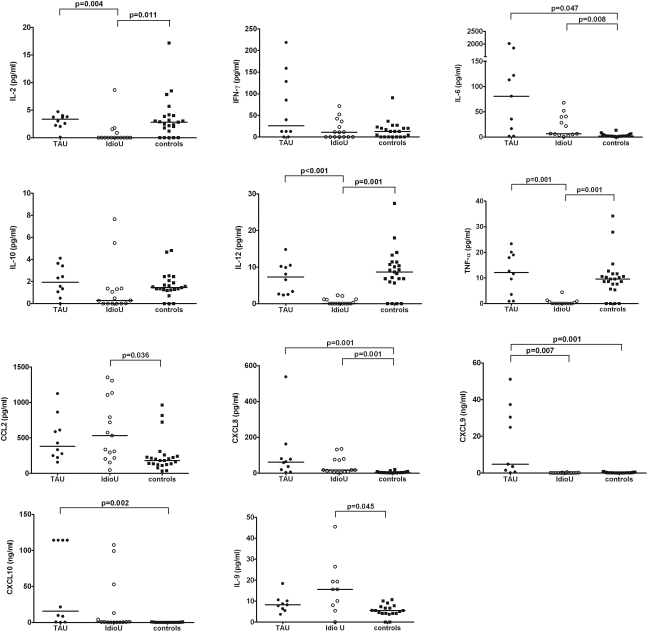
Cytokine levels measured by multiplexed bead immunoassay in aqueous from TB associated uveitis (TAU, closed circles), idiopathic uveitis (IdioU, open circles), and non-inflammatory controls (squares). Mann-Whitney U test with Bonferroni adjustment was performed to compare uveitis and control groups. A significant difference is set at p<0.05.

When the IU and cataract groups were compared using univariate analysis, IU showed significantly higher concentrations of IL-6 (p=0.008), CCL2 (p=0.036), CXCL8 (p=0.001), and IL-9 (p=0.045), and lower concentrations of IL-2 (p=0.011), IL-12 (p=0.001), and TNF-α (p=0.001), compared to the controls. After multivariate analysis adjusted for age and gender, the differences in IL-6 (p=0.050), CXCL2 (p=0.025), CXCL8 (p=0.025), IL-9 (p=0.015), and TNF-α (p=0.050) remained significant. However, the differences in IL-2 (p=0.164) and IL-12 (p=0.993) were no longer significant. Scatter plots of these cytokines are presented in Figure 1. Between the TAU and IU groups, TAU had higher IL-2 (p=0.004), IL-12 (p=0.000), TNFα (p=0.001), and CXCL9 (p=0.007) levels by univariate analysis. However, when adjusted by age and gender, only the difference in IL-12 (p=0.050) remained significant.

### Classification analysis for TAU and IU

**Figure 2:**
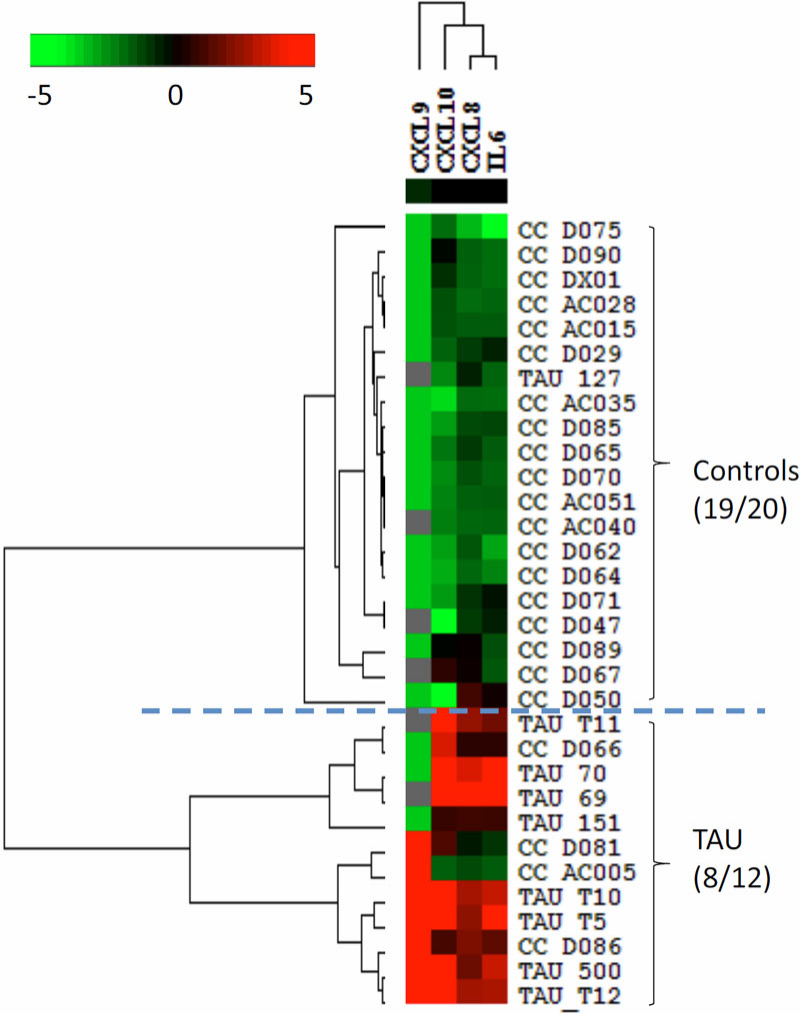
Heat-map analysis comparing uveitis associated with tuberculosis (TAU) and non-inflammatory controls. A distinct panel of cytokines composed of IL-6, CXCL8, CXCL9, and CXCL10 distinguishes both groups.

**Figure 3:**
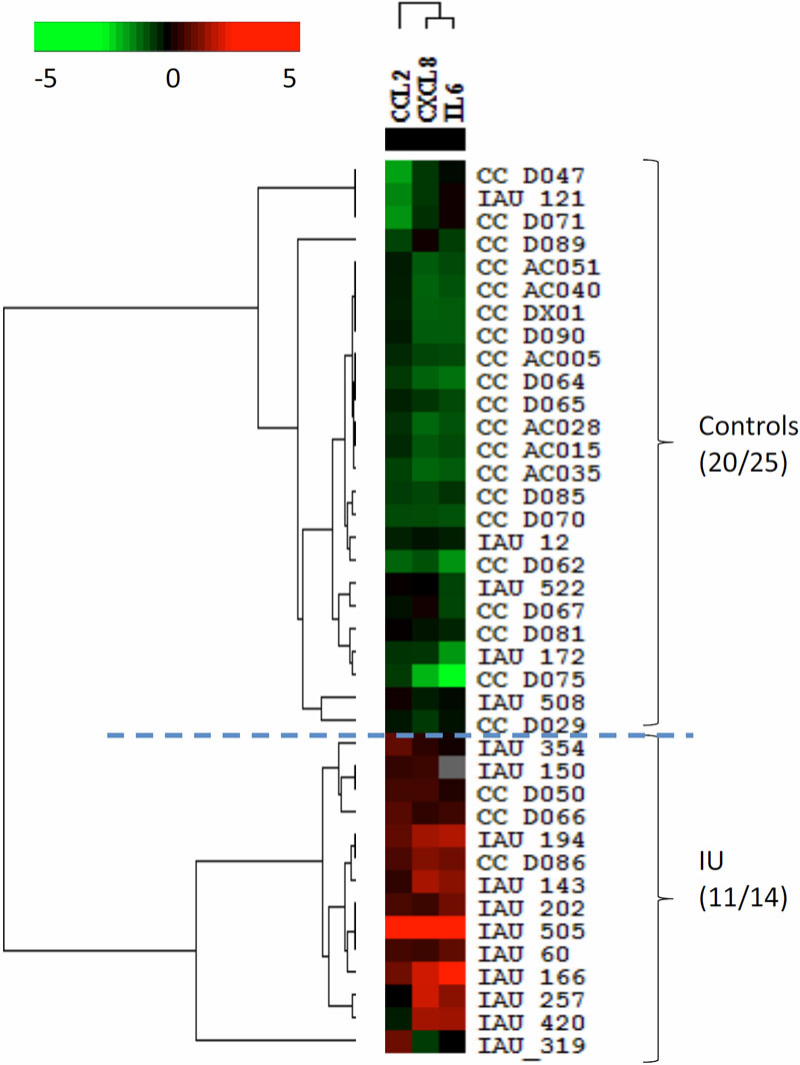
Heat-map analysis comparing idiopathic uveitis (IU) and non-inflammatory controls. The combination of CCL2, CXCL8, and IL-6 separated controls from IU with 80% accuracy.

**Figure 4:**
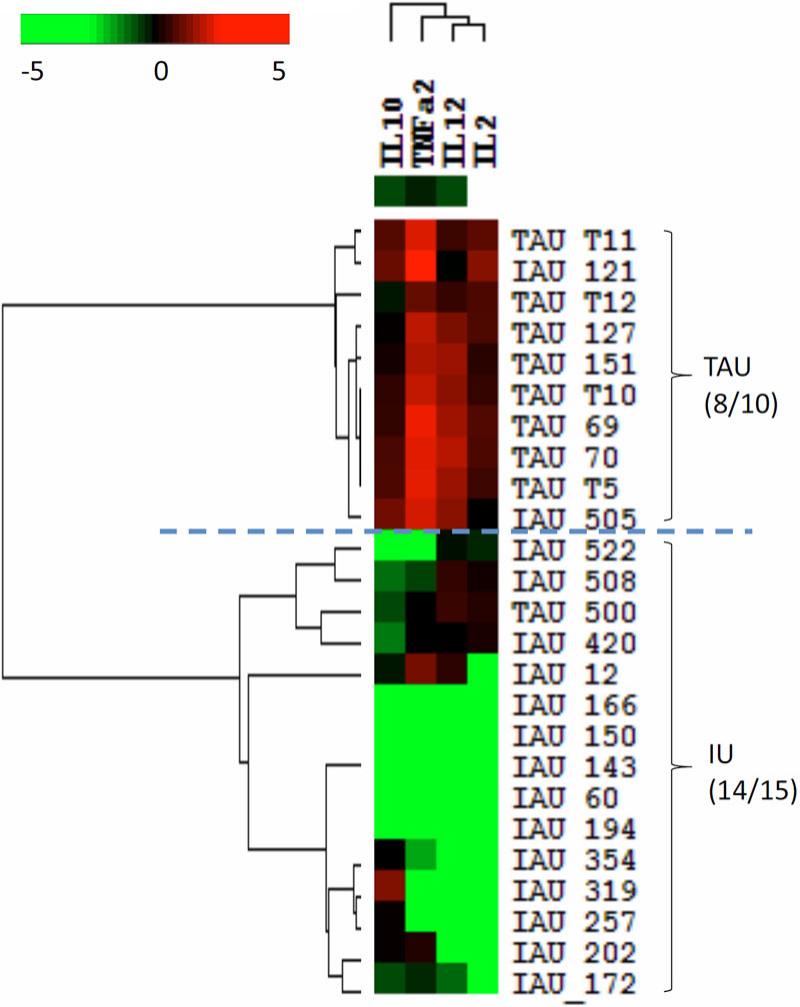
Heat-map analysis comparing uveitis associated with tuberculosis (TAU) and idiopathic uveitis (IU). The combination of IL-10, TNF-α, IL-12, and IL-2 separated TAU from IU with an accuracy of 80% for TAU and 93% for IU.

Heat map analysis revealed a distinct panel of cytokines, composed of IL-6, CXCL8, CXCL9, and CXCL10, to separate the TAU patients from the non-inflammatory controls (Figure 2). The accuracy of this classification was 95% for the non-inflammatory controls and 67% for the TAU samples. The combination of CCL2, CXCL8, and IL-6 separated the controls from the IU group, with an accuracy of 80% for controls and 80% for IU (Figure 3). The combination of IL-10, TNF-α, IL-12, and IL-2 separated the TAU group from the IU group, with an accuracy of 80% for TAU and 93% for IU (Figure 4). The decision tree analysis of all samples generated a mathematical classifier model, which involved three cytokines: IL-12, IL-6, and CCL4. A further fivefold cross validation of this model provided a correction rate of 77% for all samples tested.

## Discussion

The immune response upon active *Mycobacterium tuberculosis* infection is initiated by the recognition of the bacteria, mainly by macrophage and DC cells through Toll-like receptors, which stimulate the production of cytokines such as IL-12 and TNF-α. IL-12 subsequently activates a Th1-cell-dominated adaptive immune response, which is largely responsible for containing the inflammation, through the secretion of IFN-γ. Increased concentrations of IL-12, TNF-α, and IFN-γ are hallmarks of active TB infection. However, in our study, heat-map analysis revealed increased IL-6, CXCL8, CXCL9, and CXCL10 in the TAU group compared to the non-inflammatory controls, and it is also distinct from the cytokine profiles of idiopathic uveitis. These results suggest that TAU is a distinct entity from idiopathic uveitis, and the cytokine profiles suggest that these patients with TAU were unlikely to have active ocular TB infection.

Our heat-map analysis also revealed that CXCL9 and CXCL10 are increased in TAU, findings also seen in autoimmune-related inflammation [31,32]. A previous study described a patient who developed uveitis following *Bacille-Calmette-Guerin* therapy for bladder cancer—an autoimmune reaction against retinal proteins, which had sequences similar to *Mycobacterium tuberculosis* proteins [33]. These are consistent with previous suggestions that the ocular inflammation observed in TAU patients could be due to a mechanism similar to antigenic mimicry [25,34].

The identification of biomarkers in the aqueous humor can potentially provide us with an alternative for diagnosing conditions that may otherwise be considered as idiopathic in patients with uveitis. A previous study has shown that different cytokine profiles are associated with uveitis of specific etiologies, using multivariate analysis of aqueous cytokine composition [15]. However, multivariate analyses do not take into account the complicated intrinsic interactions between cytokines. Therefore, we used step-wise analysis and mathematical modeling with decision tree analysis, which were consistent with those that showed significant differences between groups by univariate analysis. In this study, we used tests to identify panels of cytokines that distinguish TAU from IU and non-inflammatory controls. By deleting highly correlated cytokines and cytokines that were not significantly different between groups, the analysis revealed a panel of cytokines that distinguishes samples with TAU from non-inflammatory controls. These results may be helpful in identifying key factors associated with each disease entity.

Our study is limited by the relatively small number of subjects and the cross-sectional design, in which all subjects had aqueous sampling and analysis during active disease. Although a followup aqueous sample after the treatment would give us more insightful information regarding the pathogenesis of the diseases, we did not consider it ethical to sample patients during quiescence. However, in future studies, it would be of great value to analyze the aqueous of patients with TAU who develop cataract and need to undergo surgery. Similarly, it would be ideal to sample aqueous from eyes of healthy individuals with no ocular pathology to serve as controls for future studies. We randomly recruited subjects with uveitis and non-inflammatory controls, which led to a significant difference in the mean age between groups. However, we did not find any significant changes when age and gender differences were taken into consideration using multivariate analysis.

In conclusion, our study showed that uveitis associated with TB featured increased aqueous levels of IL-6, CXCL2, CXCL8, CXCL9, and CXCL10, which is not typical of an active ocular TB infection. On the other hand, the aqueous humor cytokine profile of idiopathic uveitis suggested a dysregulated immune homeostasis, which is unlikely to be incited by a pathogen. The altered cytokine concentrations found in this study could lead to further discoveries regarding the pathogenesis of both TAU and idiopathic uveitis.
